# A Recombinant Turkey Herpesvirus Expressing the F Protein of Newcastle Disease Virus Genotype XII Generated by NHEJ-CRISPR/Cas9 and Cre-LoxP Systems Confers Protection against Genotype XII Challenge in Chickens

**DOI:** 10.3390/v14040793

**Published:** 2022-04-11

**Authors:** Katherine Calderón, Aldo Rojas-Neyra, Brigith Carbajal-Lévano, Luis Luján-Valenzuela, Julio Ticona, Gisela Isasi-Rivas, Angela Montalvan, Manuel Criollo-Orozco, Edison Huaccachi-Gonzáles, Luis Tataje-Lavanda, Karla Lucia F. Alvarez, Manolo Fernández-Sánchez, Manolo Fernández-Díaz, Na Tang, Yongxiu Yao, Venugopal Nair

**Affiliations:** 1 Research and Development Laboratories, FARVET, Carretera Panamericana Sur N° 766 Km 198.5, Chincha Alta 11702, Peru; aldo.s@farvet.com (A.R.-N.); acarbajal@farvet.com (B.C.-L.); lujanvalenzuelaluisangel@gmail.com (L.L.-V.); julio.ticona@farvet.com (J.T.); gisasi@farvet.com (G.I.-R.); angela.montalvan@farvet.com (A.M.); manuel.criollo@farvet.com (M.C.-O.); ehuaccachi@farvet.com (E.H.-G.); luistataje@farvet.com (L.T.-L.); karlalucia220@gmail.com (K.L.F.A.); manoloj@farvet.com (M.F.-S.); farvet@farvet.com (M.F.-D.); 2 Faculty of Medicine, Major National University of San Marcos, Av. Miguel Grau 755, Lima 15001, Peru; 3 Viral Oncogenesis Group & UK–China Centre of Excellence for Research on Avian Diseases, The Pirbright Institute, Pirbright, Surrey GU24 ONF, UK; tangna0543@163.com (N.T.); yongxiu.yao@pirbright.ac.uk (Y.Y.); venugopal.nair@pirbright.ac.uk (V.N.); 4 Binzhou Animal Science and Veterinary Medicine Academy & UK–China Centre of Excellence for Research on Avian Diseases, Binzhou 256600, China

**Keywords:** Newcastle disease virus, fusion gene, genotype XII, Turkey herpesvirus, CRISPR/Cas9, NHEJ, challenge protection, shed challenge virus

## Abstract

In this study, we developed a new recombinant virus rHVT-F using a Turkey herpesvirus (HVT) vector, expressing the fusion (F) protein of the genotype XII Newcastle disease virus (NDV) circulating in Peru. We evaluated the viral shedding and efficacy against the NDV genotype XII challenge in specific pathogen-free (SPF) chickens. The F protein expression cassette was inserted in the unique long (UL) UL45–UL46 intergenic locus of the HVT genome by utilizing a clustered regularly interspaced short palindromic repeat (CRISPR)/Cas9 gene-editing technology via a non-homologous end joining (NHEJ) repair pathway. The rHVT-F virus, which expressed the F protein stably in vitro and in vivo, showed similar growth kinetics to the wild-type HVT (wtHVT) virus. The F protein expression of the rHVT-F virus was detected by an indirect immunofluorescence assay (IFA), Western blotting, and a flow cytometry assay. The presence of an NDV-specific IgY antibody was detected in serum samples by an enzyme-linked immunosorbent assay (ELISA) in SPF chickens vaccinated with the rHVT-F virus. In the challenge experiment, the rHVT-F vaccine fully protects a high, and significantly reduced, virus shedding in oral at 5 days post-challenge (dpc). In conclusion, this new rHVT-F vaccine candidate is capable of fully protecting SPF chickens against the genotype XII challenge.

## 1. Introduction

Newcastle disease (ND) is a highly contagious avian disease with a significant impact on global poultry production. ND is caused by the Newcastle disease virus (NDV), formerly known as *Avian orthoavulavirus 1* (AOAV-1), belonging to the family Paramyxoviridae (https://talk.ictvonline.org/taxonomy/ accessed on 20 October 2021). The genome of NDV is single-stranded, non-segmented, negative-sense RNA encoding six structural proteins: nucleocapsid protein (NP), phosphoprotein (P), matrix protein (M), fusion protein (F), haemagglutinin–neuraminidase protein (HN), and large protein (L) [1,2]. The viral particle of NDV has the F and HN glycoproteins, both localized in the envelope of the virion, that participate in the mediation of virus attachment, entry to the cell, initiation of the infectious virus cycle, and release from the cell. The F glycoprotein has been considered as an important antigen because it provided better protection compared to HN [3].

Based on the molecular analysis, the NDV has been classified into two classes: I and II [4]. The class I isolates are all grouped into a single genotype and are generally lentogenic strains. By contrast, class II isolates are classified into genotypes I–XXI, and they can be divided into three groups according to their virulence in poultry: velogenic, mesogenic, and lentogenic [5].

The circulation of a velogenic strain of NDV, belonging to genotype XII, has been reported in South America (Colombia and Peru, subgenotype XIIa) [6,7,8]. To control its propagation, the majority of poultry farms have applied for vaccination programs, with vaccine strains belonging to genotypes I and II (i.e., LaSota and B1), which have been extensively used for years. These classical ND vaccines can be both live attenuated and inactivated, and they have demonstrated limited efficacy in controlling virus shedding and overcoming the high levels of maternally derived antibodies (MDA) that interfere with the efficacy of these vaccines in poultry.

To overcome these limitations of the classical vaccines for NDV, strategies to express immunogenic NDV proteins in live virus vectors have been explored. The most common vector vaccine used is the *Meleagrid herpesvirus 1* (MeAHV1), or Turkey herpesvirus (HVT), expressing the F or HN protein from NDV. These vectored vaccines are effective in inducing complete protection against the NDV [9,10,11,12].

The HVT, belonging to serotype 3 of Marek’s disease virus (MDV) with a double-stranded deoxyribonucleic acid (dsDNA) genome, is a nonpathogenic alphaherpesvirus of chickens [13]. HVT is widely used as a live vaccine against MDV [14], and it is considered to be a suitable vaccine candidate due to its ability to achieve persistent infection, even in the presence of MDA [10], decrease the shedding of the virus, and induce cellular and humoral immunity of a long duration (through 50 weeks of age) with a single dose vaccination that can be given in ovo or at one-day-old [10,12,15,16,17].

HVT has also been widely used as a viral vector for the expression of heterologous antigens of diverse avian diseases such as infectious bursal disease (IBD) [18], avian influenza (AI) [19,20], infectious laryngotracheitis (ILT) [21], chlamydia psittaci [22], and ND [11,23].

Several regions of the HVT genome are used for foreign gene insertion [24], and the region between UL45 and UL46 of unique long (UL) has most commonly been selected as the insertion site [20,25,26,27,28,29]. Several strategies have been used to generate recombinant HVT (rHVT), including classical homologous recombination methods, reconstitution of recombinant viral genomes from overlapping cosmids, and bacterial artificial chromosome (BAC) clones [30]. While these approaches allow the insertion of protective genes from other avian pathogens, these strategies are often time-consuming and have many limitations [26].

The clustered regularly interspaced short palindromic repeat (CRISPR)/Cas system, a new gene-editing system that was derived from a bacterial immune system, can confer the ability to remember and destroy phages as a defense tool against viral invaders [31]. The CRISPR-associated 9 (CRISPR/Cas9) type II system, consisting of RNA-guided Cas9 endonuclease from *Streptococcus pyogenes*, a single guide RNA (sgRNA), and the trans-activating crRNA (tracrRNA) [32], was developed for the editing of eukaryotic cells by introducing precise and efficient double-stranded breaks (DSBs) in the target deoxyribonucleic acid (DNA) [33]. These DSBs are subsequently repaired either through non-homologous end joining (NHEJ), which repairs the DNA while causing the presence of indels (insertions or deletions) in the region, or through homology-directed repair (HDR), which repairs the DNA in the presence of a donor with the homologous sequences to the target DNA [34]. The CRISPR/Cas9 system, which has proven to be a powerful genetic tool, has been adapted for editing DNA viruses such as the Epstein–Barr virus [35], pseudorabies virus [36,37,38,39,40,41], adenovirus [42], and vaccinia virus [43]. CRISPR/Cas9 technology has also recently been applied to edit the genome of avian herpesviruses and potential development of recombinant viral vaccines [26,27,28,29,44,45,46,47,48,49].

In this study, our main goal was to generate an rHVT vector vaccine, expressing the F protein (rHVT-F) from NDV genotype XII circulating in Peru, and evaluate the protection in specific, pathogen-free (SPF) white leghorn chickens against NDV genotype XII challenge. We chose to insert the F gene in the HVT unique long UL45–UL46 intergenic region using NHEJ-CRISPR/Cas9. The rHVT-F was evaluated, in vitro and in vivo, for the stability and integrity of the inserted cassette, as well as for the expression of the F protein. Our results confirmed, once again, that the NHEJ-CRISPR/Cas9 and Cre-LoxP systems are a fast and efficient system for editing the HVT genome, as well as an accurate pipeline for generating new recombinant viruses. The rHVT-F was evaluated as a vaccine candidate in chickens against an NDV genotype XII challenge.

## 2. Materials and Methods

### 2.1. Chickens

All procedures and experimental protocols of this study were performed following Protocol No. 01-2021, approved 12 August 2021 by the Ethics Committee of the Faculty of Veterinary Medicine and Zootechnics (FVMZ) of the National University of Hermilio Valdizan. 

One-day-old SPF white leghorn chickens from Charles Rivers Laboratory were used in this study. After hatching, all the chickens were placed in a Biosafety Laboratory 2 (BLS-2) in cages. Feed and water were provided with ad libitum access.

For the challenge, the chickens were labeled with color/number seals and transferred to a Biosafety Laboratory 3 (BLS-3) FARVET S.A.C.

Following the challenge, the chickens were monitored daily for clinical signs and mortality by 14 days. The chickens with severe clinical signs of ND were immediately euthanized/removed and counted as mortality.

### 2.2. Cell Culture and Virus

Primary chicken embryo fibroblast (CEF) cells were prepared from 10-day-old SPF embryonated chicken eggs (Charles River Avian Vaccine Services, Norwich, CT, USA) and maintained in Dulbecco’s Modified Eagle’s Medium F-12 (1:1) 1× (DMEM 1×) (catalog no. SH30004.04, HyClone, Logan, UT, USA) supplemented with 5% heat-inactivated fetal bovine serum (FBS) (catalog no. SH30066.03, Thermo Fisher Scientific, Waltham, MA, USA) and 1× antibiotic-antimycotic-100× (catalog no. 15240062, Thermo Fisher Scientific, Waltham, MA, USA) at 37 °C under a 5% CO_2_ atmosphere.

The wild-type HVT (wtHVT) Fc126 strain, obtained from Avian Disease and Oncology Laboratory (ADOL) (East Lansing, MI, USA), was used for the construction and production of the new recombinant virus.

The challenge strain NDV, known as NDV/peacock/Peru/2011 (PP2011) (GenBank accession no. KR732614) belonging to Class II genotype XII of NDV, intracerebral pathogenicity index (IPIC) 1.80, with the characteristic of pathotype velogenic, was previously isolated and characterized in Peru [7].

### 2.3. Design of sgRNAs and Construction of the Donor Plasmid

The intergenic target sequence of the UL45–UL46 region, from the HVT genome, was submitted for the design of the sgRNAs (https://www.genscript.com/gRNA-design-tool.html accessed 29 May 2019), and those with the highest scores were selected. Plasmid px459v2.0 (catalog no. 62988, Addgene, Cambridge, MA, USA) was digested with BbsI-HF (NEB, New England BioLabs, Ipswich, MA, USA) and then purified using a Gel Extraction Kit QIAquick (Qiagen, Valencia, CA, USA), according to the manufacturer’s instructions. The oligo-DNA primers sgRNA-UL45-46-F/R, corresponding to the sgRNA of the target sequence, were synthesized by Integrated DNA Technologies, Inc. (IDT, Coralville, IA, USA) and cloned into the previously digested cloning vector px459v2.0 for construction of px459v2.0-sgRNA-UL45-46. The sg-A sequence was from a previous publication [33] and cloned into px459v2.0 in the same way.

For the construction of the donor plasmid, we used the plasmid pGEM-sgA-GFP-VP2, which contained: two sg-A target sequences at both ends, two LoxP sequences for the green fluorescent protein (GFP) reporter cassette excision, and a VP2 protein expression cassette flanked with two SfiI restriction sites [27]. The pGEM-sgA-GFP-VP2 was digested by the enzyme SfiI to remove the VP2 protein expression cassette, obtaining the linearized plasmid pGEM-sgA-GFP. A new F expression cassette that contains the complete open reading frame (ORF) of the F protein, from the NDV of the genotype XII strain PP2011 (GenBank accession no. KR732614), previously isolated in Peru [7] under the control of a murine cytomegalovirus (mCMV) promoter and poly-A signal sequence (pA), was designed. This cassette was synthesized by GenScript (Piscataway, NJ, USA) and was subsequently cloned into plasmid pUC57 via SfiI restriction sites. Following this, the plasmid was digested with SfiI, and the F protein expression cassette was purified for subcloning into pGEM-sgA-GFP. The resulting plasmid was designated pGEM-sgA-GFP-F-XII. The insertion of the F expression cassette into the plasmid was verified by polymerase chain reaction (PCR) (data not shown) using specific primers 1F/1R and 2F/2R (Table 1).

### 2.4. Generation of the Recombinant HVT Containing the F Gene from NDV of Genotype XII

CEF cells, grown in 12-well plates, were transiently transfected with 1 μg donor plasmid and 0.5 μg of each sgRNA using Lipofectamine^®^ Reagent (Thermo Fisher Scientific, Waltham, MA, USA), according to the manufacturer’s protocol. At 24 h post-transfection (hpt), the culture medium was changed to DMEM 1× supplemented with 2% FBS, and wtHVT infection was performed at a multiplicity of infection (MOI) of 0.01. Three days later, the transfected/infected cells were transferred into six-well plates, and clones expressing GFP marker were then isolated through three rounds of plaque isolations by picking green fluorescence plaques. Posteriorly, single cells expressing GFP were sorted into 96-well plates by fluorescence-activated cell sorting (FACS) on a BD FACSMelody™ Cell Sorter (BD Biosciences, San Jose, CA, USA).

**Figure 1:**
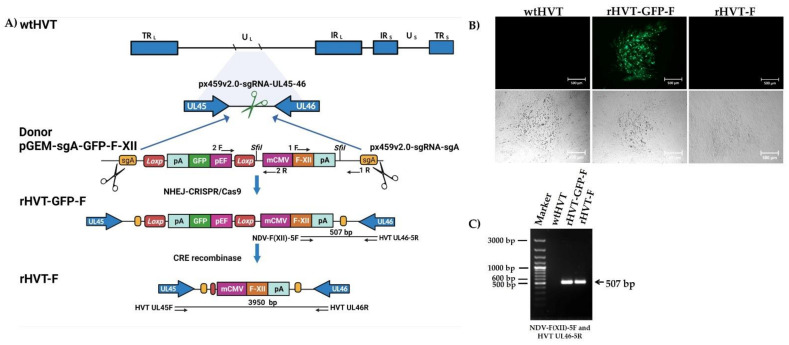
The strategy used for the generation of the rHVT-F virus. (**A**) The genome of the wild-type (wt) Herpesvirus of turkey (HVT) is shown with unique long (UL), unique short (US), terminal and internal repeat long (TRL/IRL), and short (TRS/IRS) regions. The single guide (sg)RNA-UL45-46 introduced double-stranded breaks (DSBs) in the intergenic region UL45–UL46 of the wtHVT genome. The scissor symbol (green) represents the sgRNA-UL45-46 targeting site in the UL45-UL46 of the wtHVT genome. The green fluorescence protein (GFP) expression cassette contains: the elongation factor promoter (pEF), the complete open reading frame (ORF) of the GFP, and poly-A signal sequence (pA); Fusion (F) expression cassette contains: murine cytomegalovirus promoter (mCMV), the ORF of the F protein of Newcastle disease virus (NDV), and pA. The GFP and F expression cassettes from the donor plasmid were flanked with sg-A target sites to introduce the desired cleavage for its release and integration using the non-homologous end joining (NHEJ)—clustered regularly interspaced short palindromic repeat (CRISPR) -associated 9 (CRISPR/Cas9) strategy into the genome of wtHVT. The scissor symbol (blacks) represents the sgRNA-sgA targeting sites in the donor plasmid. The GFP reporter expression cassette was excised from rHVT-GFP-F using the Cre-loxP System and the new recombinant HVT was named rHVT-F. The fragments amplified by polymerase chain reaction (PCR) using the primers 1F/1R and 2F/2R showed the correct insertion of the F expression cassette into plasmid pGEM-sgA-GFP-F-XII. The junction primers NDV-F(XII)-5F/HVT UL46-5R (507 bp, base pairs = bp) showed the insertion of the F expression cassette at the correct locus into the genome rHVT-GPF-F and rHVT-F (after excision of the GFP reporter cassette). The primers HVT UL45F/HVT UL46R (3950 bp) showed the presence of the complete NDV F expression cassette in the genome at the correct locus of rHVT-F. This drawing was created with BioRender.com (Not drawn to scale). (**B**) Comparison of plaque morphology between rHVT-GFP-F, rHVT-F, and wtHVT. The plaque morphology of infected chicken embryo fibroblast (CEF) cells, induced by the rHVT-GFP-F, rHVT-F, and wtHVT viruses, was observed by fluorescence (top panel) and bright-field (bottom panel) microscopy: 100× magnification, and all scale bars represent 500 µm. (**C**) The presence of the NDV F expression cassette in the HVT genome was verified by PCR using the primers NDV-F(XII)-5F and HVT UL46-5R, as shown in (**A**) in both rHVT-GFP-F and rHVT-F, respectively.

The insertion of the NDV F expression cassette into the HVT genome, at the correct locus, was verified by PCR using junction primers NDV-F(XII)-5F and HVT UL46-5R (Table 1). Correct knock-in of the cassette was confirmed from the sequence of the PCR products (Figure 1C). The new recombinant HVT was named rHVT-GFP-F.

### 2.5. Flow Cytometry Analysis

The expression of the F protein on the cell surface over time was evaluated by flow cytometry. CEF cells grown in a T75 flask were infected, at an MOI of 0.01, with the rHVT-GFP-F virus and maintained for 48 to 72 h before harvesting. After washing the cells twice with 5% FBS in Phosphate-buffered saline 1× (PBS 1×), non-specific antibody sites were blocked with 5% Normal Mouse Serum (catalog no. ab7486, Abcam, Cambridge, MA, USA) in FACS Buffer for 15 min at 4 °C. The cells were then incubated with anti-NDV chicken serum (#10100482, Charles River Avian Vaccine Services, Norwich, CT, USA) (1:6400) for 2 h at 4 °C. After washing twice with 5% FBS in PBS 1×, the cells were incubated with 2.5 µg/mL goat anti-chicken IgY conjugated with Alexa Fluor^®^ 647 (catalog no. ab150171, Abcam, Cambridge, MA, USA) for 30 min at 4 °C. The cells were then washed twice and resuspended in 5% FBS in PBS 1×. Flow cytometry was performed (Beckman Coulter Gallios, Brea, CA, USA) in a 3 laser/10 color configuration, and the data were analyzed using FlowJo software v7.6.5 (TreeStar, Ashland, OR, USA). The results are expressed as the percentage of infected cells (%). The expression level of the protein is reported as the median fluorescence intensity (MFI). For each sample, 30,000 events were acquired. The experiment was independently repeated three times.

### 2.6. Excision of the GFP Reporter Cassette Via the Cre-LoxP System

The GFP reporter expression cassette was excised upon Cre recombinase. CEF cells were seeded in 6-well plates and then infected with rHVT-GFP-F, with an MOI of 0.001. After 24 h post-infection (hpi), the cells were transfected with 2 µg of the Cre recombinase plasmid pcDNA3-Cre [26]. Four days later, GFP-negative plaques were selected and purified by three rounds of plaque picking. The complete excision of the GFP cassette was evaluated by PCR, using specific primers HVT UL45F and HVT UL46R (Table 1). The presence of the NDV F expression cassette in the HVT genome at the correct locus was verified, again, by PCR using junction primers NDV-F(XII)-5F and HVT UL46-5R (Table 1). The resulting recombinant HVT was named rHVT-F.

### 2.7. Indirect Immunofluorescence Assay (IFA)

CEF cells, grown in 12-well plates, were infected at an MOI of 0.005 with the wtHVT or the rHVT-F virus. At 72 hpi, the cells were washed three times with Dulbecco’s phosphate-buffered saline 1× (DPBS 1×), fixed with 4% paraformaldehyde for 15 min, and permeabilizated with 0.1% TRITON^®^ X-100 (catalog no. 648463, Merck, Darmstadt, Germany) in DPBS 1× for 10 min at room temperature. Cells were incubated with the primary antibody in a solution of 5% bovine serum albumin (BSA) (catalog no. A7030-500G, Sigma-Aldrich, St. Louis, MO, USA) in DPBS 1× for 1 h at room temperature. A mouse antibody against MDV (B149M) (1:200) (catalog no. ab90487, Abcam, Cambridge, MA, USA) was used as the positive control for the detection of HVT infection, and anti-NDV chicken serum (1:200) (#10100486, Charles River Avian Vaccine Services, Norwich, CT, USA) was used for the detection of the F protein expression. After washing three times with DPBS 1×, the cells were incubated with goat anti-mouse IgG H&L Alexa Fluor^®^ 594 (1:100) (catalog no. ab150116, Abcam, Cambridge, MA, USA) and Goat anti-chicken IgY H&L Alexa Fluor^®^ 405 (1:200) (catalog no. ab175674, Abcam, Cambridge, MA, USA), respectively, in DPBS 1× with 5% BSA for 45 min at room temperature. The results were observed using an Axio ObserverA1 fluorescence microscope (Carl Zeiss, Jena, Germany). Digital images were taken at 50× magnification and processed using an AxioCam MRc5 camera (Carl Zeiss, Jena, Germany).

### 2.8. Western Blot Analysis

To evaluate NDV F protein expression by the rHVT-F virus, CEF cells were infected with the recombinant virus at an MOI of 0.01. At 72 hpi, the CEF cells were lysed and analyzed by Western blotting. Additionally, Western blot analysis was carried out to examine the incorporation of the F protein into the recombinant virus particles. For this, viruses from the supernatants of CEF cells infected with the recombinant virus were concentrated through tangential filtration using Sartoflow Advanced (Sartorius, Gottingen, Germany) and subjected to ultracentrifugation in an Optima L-100K Ultracentrifuge (Beckman Coulter Inc., Brea, CA, USA) using sucrose gradients to obtain partially purified virus particles. Western blot analysis was carried out using a specific rabbit polyclonal antibody against NDV F protein (20:5000) (GenScript Laboratories, Piscataway, NJ, USA) as a primary antibody and a mouse anti-rabbit IgG antibody, conjugated to horseradish peroxidase (HRP) (2:5000) (catalog no. A01827, GenScript Laboratories, Piscataway, NJ, USA), as a secondary antibody. Simultaneously, the detection of the Beta-actin protein, as a loading control in cell lysates, was carried out using a mouse monoclonal antibody to Beta-actin (catalog no. ab8226, Abcam, Cambridge, MA, USA) (5:5000) as the primary antibody and goat polyclonal antibody anti-mouse IgG, conjugated to HRP (catalog no. A00160, GenScript Laboratories, Piscataway, NJ, USA) (3:5000), as the secondary antibody. The protein expression was visualized with a CCD camera Azure c600 imaging system (Azure Biosystems, Inc., Dublin, OH, USA).

### 2.9. In Vitro Growth Properties and Plaque Assay

The viral titers were determined by plaque-forming units (PFUs) in CEF cells. To compare the infectivity and growth properties of the wtHVT and the rHVT-F virus, CEF cells grown in 6-well plates were infected in duplicate, with 100 PFUs per well of each virus, and harvested at 24, 48, 72, 96, and 120 hpi. The harvested cells were titrated by plaque assay using CEF cells seeded in 6-well plates at 50% confluency. The PFUs of the different dilutions were determined and counted five days later using an Axio Observer.A1 inverted fluorescence microscope (Carl Zeiss, Jena, Germany). The experiment was independently repeated three times.

### 2.10. Genetic Stability of the rHVT-F Virus

To evaluate the genetic stability of the rHVT-F virus stocks, they were sequentially grown on CEF cells for 20 passages. The viral DNA was extracted from the infected CEF cells at every fifth passage, and the presence and stability of the inserted cassette were confirmed by PCR using specific primers HVT UL45F and HVT UL46R (Table 1). The expression of the F protein was also evaluated at 20 passages by IFA, as described above. The experiment was independently repeated three times.

### 2.11. Vaccination in SPF Chickens and Efficacy of the rHVT-F Vaccine against NDV Genotype XII Challenge in SPF Chickens

To assess the replication and stability of rHVT-F virus in vivo, ten 1-day-old SPF chickens were vaccinated with 2500 PFUs of rHVT-F or wtHVT viruses (*n* = 5, chickens respectively).

Furthermore, to evaluate the immunogenicity and efficacy of the rHVT-F virus, twenty-one 1-day-old SPF white leghorn chickens were randomly divided and distributed into two groups: Group #1 = vaccinated with rHVT-F virus (*n* = 14) and group #2 = non-vaccinated control to challenge (*n* = 7). Group #1 was vaccinated with 2500 PFUs/chicken (in a commercial volume of 200 µL) by subcutaneous injection in the neck. Serum antibodies were tested by enzyme-linked immunosorbent assay (ELISA) and a hemagglutination-inhibition (HI) test.

To determine the efficacy of the rHVT-F vaccine against NDV genotype XII, group #1 = vaccinated with rHVT-F virus and group #2 = non-vaccinated were challenged at 49 dpv with the velogenic NDV genotype XII, NDV/peacock/Peru/2011 (PP2011) strain. The challenge virus was administrated (in 0.05 mL) by the oculo-nasal route with 10^5^ median lethal doses (LD_50_) that induce 100% mortality within 4–6 days in SPF chickens (data not reported). Following the challenge, chickens were monitored daily for overt clinical signs and mortality by 14 days. Oral and cloacal swabs were collected for the quantification of challenge virus shedding by plaque assay. Finally, the protection was determined based on mortality and the presence of clinical signs of ND.

### 2.12. ELISA and HI Test

To measure the specific immunoglobulin Y (IgY) antibodies against NDV, the sera from blood samples were collected partially from chickens, via wing-web bleeding, at 20, 34, and 49 days post-vaccination (dpv). All serum samples were separated from blood samples by centrifugation at 3500 rpm for 7 min at room temperature. For the ELISA, the commercial kit ID Screen^®^ Newcastle Disease Indirect (ID.Vet, Grabels, France) was used following the manufacturer’s protocol. The reading was made at 450 nm using an automated microplate reader (BioTek Instruments Inc., Winooski, VT, USA). All serum samples were analyzed using a positive and negative control antisera provided by the kit, and the antibody titers were quantified using the software provided by the manufacturer. The serum samples with the ELISA antibody titer ≥ 993 were considered positive. The HI was only evaluated at 49 dpv; the antibody titers were calculated using 4 haemagglutination units (HU) of the LaSota strain as an antigen, following the procedure described by World Organization for Animal Health (OIE) Terrestrial Manual-Chapter 3.3.14. Newcastle Disease (Infection with Newcastle Disease Virus) [50].

### 2.13. Evaluation of Challenge Virus Shedding

Oral and cloacal swab samples were collected from chickens at 3, 5, and 8 days post-challenge (dpc). These samples from group #1 (*n* = 7) and group #2 (*n* = 7) were quantified by a plaque assay. The swabs were soaked in 1 mL of DPBS 1× with 10× antibiotic-antimycotic-100× (catalog no. 15240062, Thermo Fisher Scientific, Waltham, MA, USA) for 30 min at 4 °C, and then, they were centrifuged at 5000 rcf for 15 min at 4 °C to clarify. Then, 25 µL of supernatants from each swab were serially diluted in 10-fold dilutions in 225 µL of serum-free DMEM 1× 200 µL of each dilution were added into each well of the DF-1 monolayer previously seeded in 12-well plates and incubated for 1 h of adsorption at 37 °C under a 5% CO_2_ atmosphere, and DMEM 1×, supplemented with 0.5% agarose, 2% FBS, and 30 mM MgCl_2_, were placed on the cell monolayer. Four days after, the cells were fixed and stained with 3.2% of paraformaldehyde and 0.2% of crystal violet. The NDV titers were reported as PFU/mL.

### 2.14. Replication and Stability of rHVT-F Virus In Vivo

The virus isolation was performed using peripheral blood lymphocytes (PBLs) collected from chickens vaccinated with the rHVT-F and wtHVT. Heparinized blood samples (pool *n* = 5 chickens per each virus) were collected at 28, 35, and 74 dpv. Among those, 4 ml of the pool from blood samples were diluted with an equal volume of DPBS 1× at room temperature and were homogeneous by inversion for three times. Additionally, 4 mL from blood/DPBS 1× mixture was placed over a layer of 4 mL of Histopaque^®^-1077 (catalog no. 10771, Sigma-Aldrich, St. Louis, MO, USA) (1:1). The samples were centrifuged at 400 rcf for 30 min at room temperature. The lymphocytes were collected from the interface between the histopaque and serum, then washed twice with DPBS 1× at 300 rcf by 10 min. Finally, the lymphocytes were resuspended in DMEM 1× supplement with 10% SFB and inoculated into a CEF cells monolayer and co-cultured until plaques were observed. The presence of HVT and stability of the NDV F protein expression in rHVT-F virus at 28, 35, and 74 dpv, was assessed by IFA, PCR, and Western blot analysis described previously.

### 2.15. Statistical Analysis

All statistical analyses were performed using GraphPad Prism v8.0.1 (GraphPad Software, San Diego, CA, USA). The differences in antibody titers, between vaccinated and non-vaccinated groups, were compared by ANOVA Tukey’s multiple comparisons test within each day. The Mann–Whitney test was used to compare the differences of viral shedding between each group within each day and type of samples. Mantel–Cox test was used to compare the survival between groups. Differences were considered significant at * *p* < 0.05, ** *p* < 0.01, *** *p* < 0.001, and **** *p* < 0.0001.

## 3. Results

### 3.1. Construction and Rescue of the rHVT-F Virus

The sgRNAs (sgRNA-sgA and sgRNA-UL45-46) were designed, synthesized, and cloned into the px459v2.0 plasmid, which contains Cas9 gene from *S. pyogenes*. The resulting plasmids were named px459v2.0-sgRNA-sgA and px459v2.0-sgRNA-UL45-46.

The donor plasmid pGEM-sgA-GFP-F-XII generated from pGEM-sgA-GFP-VP2, carried the GFP reporter expression cassette flanked with two LoxP sequences for excision and F expression cassette. The two cassettes were flanked by the sg-A target sites to introduce the desired cleavage for its release and integration into the UL45–UL46 intergenic region of the wtHVT genome, following the introduction of a DSB. The cleavage of the targeted viral genome and the donor plasmid, by Cas9, promoted the insertion of the GFP and F expression cassettes into the UL45–UL46 intergenic region of the wtHVT genome, as shown in Figure 1A. The successful insertion of the GFP and F expression cassettes into the genomic region showed that NHEJ-mediated repair allows the knock-in of the expression cassettes after the introduction of a DSB in the HVT genome by CRISPR/Cas9.

For the generation of the rHVT-GFP-F virus, CEF cells were co-transfected with pGEM-sgA-GFP-F-XII, px459v2.0-sgRNA-sgA, and px459v2.0-sgRNA-UL45-46. At 24 hpt, the cells were infected with the wtHVT at an MOI of 0.01. The plaques expressing GFP were visible at about three days post-transfection (dpt), demonstrating the successful insertion of the donor template (Figure 1B). The recombinant virus was isolated through three rounds of plaque isolation by picking the green fluorescent plaques, after which single cells expressing GFP were sorted into 96-well plates by FACS. The presence of the F expression cassette into the UL45–UL46 intergenic region was confirmed by PCR using the junction primers NDV-F(XII)-5F and HVT UL46-5R, which showed correct insertion into the wtHVT genome (Figure 1C).

Posteriorly, the excision of the GFP reporter expression cassette was carried out by Cre recombinase treatment (Cre-LoxP System), and it was confirmed by PCR using primers HVT UL45F and HVT UL46R (Figure 1A). The presence of the NDV F expression cassette into the wtHVT genome at the correct locus was detected, again, by PCR using junction primers NDV-F(XII)-5F and HVT UL46-5R (Figure 1C). The new recombinant HVT was named rHVT-F.

### 3.2. Fusion Protein Expression on the Cell Surface by Flow Cytometry Analyses

**Figure 2:**
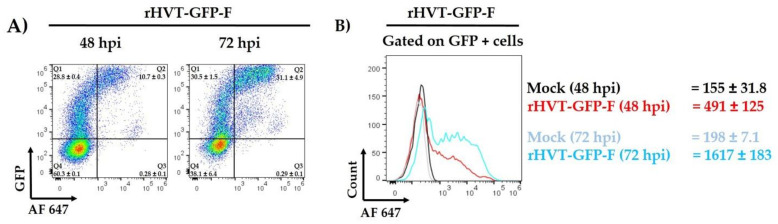
Evaluation of the cell surface expression of the F protein and its expression over time. The cells were infected with the rHVT-GFP-F virus at a multiplicity of infection (MOI) of 0.01 and harvested at 48 and 72 h post-infection (hpi) to evaluate the cell surface expression and overall expression levels of the F protein. The CEF cells were labeled with anti-NDV chicken serum as the primary antibody and Alexa Fluor^®^ 647-labeled anti-chicken IgY as a secondary antibody. (**A**) Percentage of infected cells expressing the GFP (Q1) or GFP and F protein (Q2). (**B**) The F protein expression was evaluated by determining the median fluorescence intensity (MFI) and gated in the GFP+ population. The MFI of cells infected with the rHVT-GFP-F at 48 hpi is shown in red, and at 72 hpi is in light blue. Black and blue correspond to the MFI of uninfected cells (Mock) from 48 to 72 hpi, respectively. The cells were assessed by flow cytometry (Beckman Coulter Gallios, CA, USA) in a 3 laser/10 color configuration, and the data were analyzed using FlowJo software v7.6.5 (TreeStar, Ashland, OR, USA). For each sample, 30,000 events were acquired. Data are shown as mean ± standard deviation (SD) of three independent experiments, but only a representative image is shown.

For this assay, the rHVT-GFP-F virus containing the GFP expression cassette was used. To evaluate the expression of the F protein on the cell surface during the infection process, CEF cells infected at an MOI of 0.01 with the rHVT-GFP-F virus were harvested at 48 and 72 hpi to be analyzed by flow cytometry. At 48 hpi, we observed that 39.3 ± 0.63% (mean ± SD) of cells expressed the GFP protein, and 10.7 ± 0.3% of cells co-expressed the F protein. While at 72 hpi, the percentage of GFP+ cells increased to 61.95 ± 6.43%, and the cells that co-expressed the F protein increased to 31.1 ± 4.9% (Figure 2A). The expression level of the F protein was also evaluated by determining the MFI. As expected, the MFI at 72 hpi (1617 ± 183) was higher than that at 48 hpi (491 ± 125) (Figure 2B). These results indicated that the cells infected with the recombinant virus expressed the NDV F protein on their cell surface, and the percentage of cells that express this protein increased over time.

### 3.3. Characterization of the rHVT-F Virus

**Figure 3:**
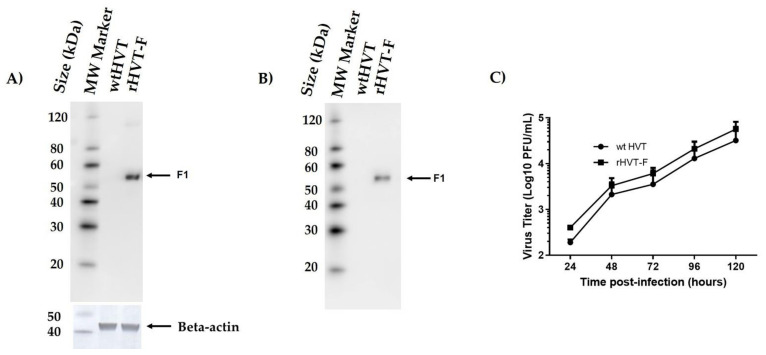
Characterization of the rHVT-F. Expression of the NDV F protein in CEF cells and their incorporation into rHVT-F virus particles. CEF cells were infected with the recombinant virus at an MOI of 0.01. Then, the CEF cells were harvested at 72 hpi and processed to prepare cell lysates. Additionally, viruses from the supernatants of CEF cells infected with the recombinant virus were concentrated using tangential filtration on Sartoflow Advanced and subjected to ultracentrifugation on sucrose gradients to obtain partially purified virus particles. These samples were evaluated by Western blot analysis using a specific rabbit polyclonal antibody, against the NDV F protein, as primary antibody and a mouse anti-rabbit IgG antibody, conjugated to horseradish peroxidase (HRP), as a secondary antibody. (**A**) From the left to the right are molecular weight (MW) markers (lane 1), cell lysates from wtHVT-infected CEF cells (lane 2), and rHVT-F-infected CEF cells (lane 3). The Beta-actin protein (~42 kDa) was used as a loading control in cell lysate. (**B**) From the left to the right are MW markers (lane 1), partially purified virus particles from the supernatant: of wtHVT-infected CEF cells (lane 2), and rHVT-F-infected CEF cells (lane 3). The black arrow indicates a ~52 kDa band corresponding to the cleaved subunit (F1) of the NDV F protein. (**C**) In vitro replication kinetics of the rHVT-F virus. CEF cells were infected with the rHVT-F virus and wtHVT at 100 plaque-forming units (PFUs). The infected cells were harvested at 24, 48, 72, 96, and 120 hpi and titrated by the plaque assay. Then, the viral titers were analyzed on two-way ANOVA with multiple comparison tests. The results were not statistically significant (*p* > 0.05).

To further confirm the expression of the inserted F in rHVT-F virus-infected CEF cells, Western blotting was performed using a specific rabbit polyclonal antibody against NDV F protein. As expected, a protein band with an apparent molecular mass of ~52 kDa, corresponding to the cleaved subunit (F1) of the NDV F protein, was detected in cell lysates from CEF cells infected with the recombinant virus. The Beta-actin protein of ~42 kDa was detected as a loading control in cell lysate (Figure 3A). To examine the incorporation of the F protein into the recombinant virus particles, the virus was partially purified from the supernatant of infected CEF cells, by sucrose gradient ultracentrifugation, and analyzed by Western blotting. Similarly, the F protein was detected in the purified recombinant virus (Figure 3B), indicating that the F protein was incorporated into the viral particles. Conversely, F protein was not detected in the wtHVT-infected CEF cells or purified wtHVT particles.

The replication properties of the rHVT-F and wtHVT viruses in CEF cells were assessed by observing virus plaque morphology and the multistep growth kinetics. CEF cells grown in six-well plates were infected with 100 PFUs of the viruses. The cells were harvested at different time points and titrated by plaque assay, as previously described. These studies showed that the viral replication kinetics did not show significant differences between wtHVT and rHVT-F viruses (*p* > 0.05) (Figure 3C). Likewise, the plaque morphology was very similar to that of wtHVT (Figure S1 in Supplementary Materials).

### 3.4. Genetic Stability of the rHVT-F Virus

**Figure 4:**
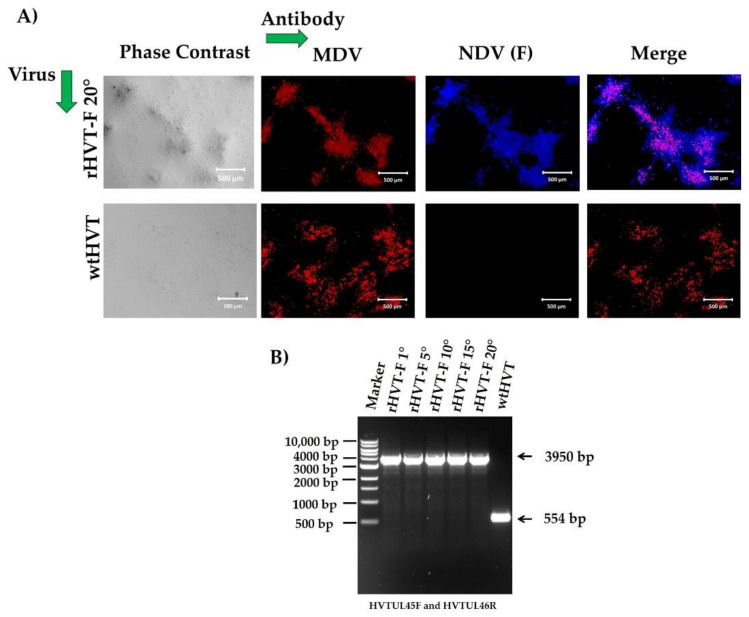
Stability of the rHVT-F virus. (**A**) The F protein expression was detected by indirect immunofluorescence assay (IFA) in the rHVT-F virus-infected CEF cells at 20th passage by staining with anti-NDV chicken serum, followed by Goat anti-chicken IgY H&L Alexa Fluor^®^ 405 (blue fluorescence). HVT was detected with a mouse antibody against Marek’s disease virus (MDV) (B149M), followed by goat anti-mouse IgG H&L Alexa Fluor^®^ 594 (red fluorescence) and visualized by fluorescence and bright-field microscopy: 50× magnification, all scale bars represent 500 µm. (**B**) The genetic stability of rHVT-F virus was detected from infected CEF cells, so viral deoxyribonucleic acid (DNA) was extracted and analyzed by PCR with primers HVT UL45F and HVT UL46R at passage 1, 5, 10, 15, and 20. The primer sequences and direction are shown in Figure 1A. The molecular sizes are indicated to the left of the gel in bp.

IFA showed the expression of the F protein in the recombinant-virus-infected CEF cells at the 20th passage (Figure 4A). These observations demonstrated that the replication of the recombinant virus did not affect the expression of the NDV F protein. To determine the genetic stability of the rHVT-F virus, the virus was grown, sequentially, on CEF cells for 20 passages. The viral genome, analyzed after every five passages by PCR, showed a 3950 bp band and did not show the appearance of the band of 554 bp, which is only present in wtHVT, suggesting stability of the insert in the recombinant viral genome (Figure 4B).

### 3.5. Humoral Immune Response to Vaccination in SPF Chickens

**Figure 5:**
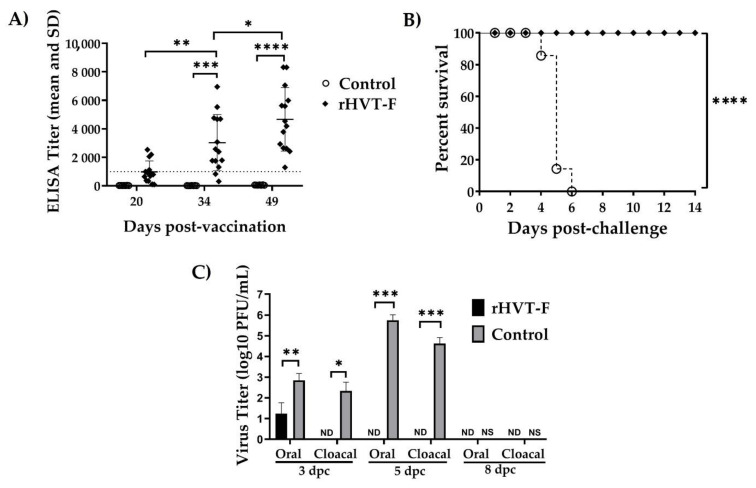
Humoral immune response and protection conferred by rHVT-F vaccine against genotype XII NDV challenge. Two groups of specific pathogen-free (SPF) chickens: Group #1 = vaccinated with rHVT-F virus (*n* = 14) and group #2 = non-vaccinated control to challenge wtHVT (*n* = 7) were immunized once at 1-day-old, and the challenge performed 49 days post-vaccination (dpv) with 10^5^ median lethal doses (LD_50_)/chicken via the oculo-nasal route. (**A**) NDV-specific IgY antibodies in vaccinated chickens at 20, 34, and 49 dpv. Serum samples that were negative for enzyme-linked immunosorbent assay (ELISA) titer are shown below the limit of detection (Cutoff = 993). SD: Standard Deviation. (**B**) The survival rate of vaccinated chickens showed no mortality in 14 days following the challenge (dpc). (**C**) Titers of challenge virus shedding, from oral and cloacal swabs from chickens, after the challenge is quantitated by a plaque assay. ND = not detected; NS = no survivors; dpc = days post-challenge. * *p* < 0.05, ** *p* < 0.01, *** *p* < 0.001, and **** *p* < 0.0001.

The serological monitoring showed that the SPF chickens from the group vaccinated with the rHVT-F vaccine developed specific IgY antibodies response against NDV detected by ELISA. The proportion of positive chickens was 28.6% at 20 dpv, with titers of 972 ± 769.5 (mean ± SD); then, the seropositivity increased to 85.7%, with titers of 3025 ± 1975.5 at 34 dpv; finally, all chickens were positive (to 100%) at 49 dpv, with titers of 4664 ± 2231.1. The results indicated that rHVT-F vaccine induced a significant increase in antibody response at 34 (*p* = 0.0063) and 49 dpv (*p* = 0.0497) (Figure 5A). The ELISA showed negative results in all the non-vaccinated control chickens at 20, 34, and 49 dpv, with titers of 16 ± 6.4, 9 ± 7.2, and 46 ± 13.4 (mean ± SD), respectively, that were far below the positivity limit of 993.

The antibody level by HI test was measured at 49 dpv only, where all serum samples collected from vaccinated chickens were positive by HI, and the mean titer was 4.3 ± 1.2 log2. Therefore, the rHVT-F virus was able to elicit an efficient humoral immune response against NDV in SPF chickens with only one dose vaccination.

### 3.6. Efficacy against Genotype XII NDV Challenge

The protective efficacy in vaccinated chickens was demonstrated by the absence of clinical signs and mortality during the 14 dpc. One-day-old SPF chickens were vaccinated with a single dose of the rHVT-F vaccine, by the subcutaneous route in the neck, and then challenged at the 49 dpv with genotype XII NDV by the oculo-nasal route. All the chickens vaccinated with rHVT-F vaccine showed complete protection against the genotype XII NDV challenge, resulting in 100% protection (14/14) and free of clinical signs. By contrast, the non-vaccinated chickens in the control group presented typical NDV clinical signs between days 3 to 5 dpc and reaching 100% (7/7) mortality at 6 dpc (Figure 5B).

The shedding of the challenge virus was quantified from oral and cloacal swab samples by the plaque assay in DF-1 cells with the appearance of large size plaques on 4 days post-assay. There was a significant effect and strong reduction in the titers of the virus shedding from the upper tract respiratory (from oral swab samples) from vaccinated chickens compared to the non-vaccinated control group after the challenge, where five out of seven chickens were negative at 3 dpc (*p* = 0.0012), and seven out of seven chickens were negative at 5 (*p* = 0.0006) and 8 dpc. Compared to this, the non-vaccinated and challenged control group, all the chickens were positive from oral swab samples at 3 and 5 dpc (Table 2 and Figure 5C). Chickens from the non-vaccinated group showed high titers of infectious virus shed on day 5 dpc.

The difference in titers of challenge virus shedding between the vaccinated and the non-vaccinated groups was even higher from the cloacal swab samples, as no viral shedding was detectable in the digestive tract in vaccinated chickens (7 out of 7 chickens were negative at 3, 5, and 8 dpc). At the same time, a significant amount of the challenge virus could be detected in the non-vaccinated group compared to the vaccinated group at 3 (*p* = 0.0210) and 5 dpc (*p* = 0.0006) from cloacal swab samples (Table 2 and Figure 5C).

### 3.7. Detection of the Replication by rHVT-F Virus Isolation from Lymphocytes

The replication was evaluated based on the viral isolation, in CEF cells, of rHVT-F and wtHVT viruses from the lymphocytes of vaccinated chickens. The appearance of a cytopathic effect, its morphology, and size in CEF cells, after 7 to 10 days post-incubation of lymphocytes, was confirmed and interpreted as a positive sample to isolation. All samples collected from chickens immunized with rHVT-F and wtHVT viruses were positive at 28, 35, and 74 dpv by PCR, indicating that the insertion of the F gene into genome wtHVT did not alter the in vivo replication. No viral plaque was detected from the non-vaccinated control group interpreted as negative to isolation.

**Figure 6:**
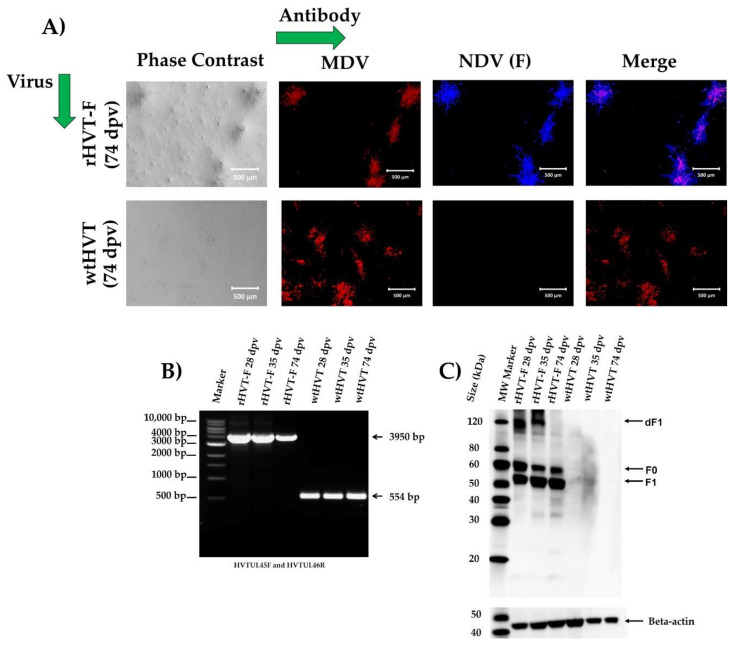
Detection of the replication and stability of rHVT-F virus in vivo. The isolation of rHVT-F and wtHVT from lymphocytes was confirmed by IFA, PCR, and Western blot analysis at 28, 35, and 74 dpv. A pool of peripheral blood lymphocytes (PBLs) from chickens (*n* = 5) was used to isolate each virus, but only a representative image from each virus is shown. (**A**) The IFA was made in CEF cells infected with the isolated viruses by staining with anti-NDV chicken serum, followed by goat anti-chicken IgY H&L Alexa Fluor^®^ 405 (blue fluorescence). HVT was detected with a mouse antibody against MDV (B149M), followed by goat anti-mouse IgG H&L Alexa Fluor^®^ 594 (red fluorescence) and visualized by fluorescence and bright-field microscopy: 50× magnification, all scale bars represent 500 µm). (**B**) The genetic stability of isolated viruses was detected from infected CEF cells, so viral DNA was extracted and analyzed by PCR with primers HVT UL45F and HVT UL46R. From the left to the right are MW marker (lane 1), extracted DNA from CEF cells infected with the isolated viruses: rHVT-F virus at 28 (lane 2), 35 (lane 3), 74 dpv (lane 4), as well as wtHVT virus at 28 (lane 5), 35 (lane 6), 74 dpv (lane 7). The molecular sizes are indicated to the left of the gel in the bp. The black arrow indicates the MW expected of the band. (**C**) Expression of the NDV F protein was evaluated by Western blot analysis in CEF cells infected with isolated viruses using a specific rabbit polyclonal antibody against the NDV F protein as a primary antibody and a mouse anti-rabbit IgG antibody conjugated to HRP as a secondary antibody. From the left to the right are MW marker (lane 1), cell lysates from CEF cells infected with the isolated viruses: rHVT-F virus at 28 (lane 2), 35 (lane 3), 74 dpv (lane 4) and wtHVT virus at 28 (lane 5), 35 (lane 6), 74 dpv (lane 7). The black arrow indicates a ~59 kDa band corresponding to the inactive precursor (F0), a band ~52 kDa corresponding to cleaved subunit (F1), and a band ~120 kDa corresponding to the F1 dimer (dF1) of the NDV F protein. The Beta-actin protein (~42 kDa) was used as a loading control in lysate cells.

The expression stability of the F gene was confirmed from the first isolation to the last viral isolation by IFA (Figure 6A), PCR (Figure 6B), and Western blot analysis (Figure 6C), indicating that expression is stably maintained in vivo. The Western blot analysis showed a ~59 kDa band corresponding to the inactive precursor (F0), a band ~52 kDa corresponding to the cleaved subunit (F1), and a band ~120 kDa corresponding to the F1 dimer (dF1) of the NDV F protein (Figure 6C). The plaque morphology and expression from HVT and F protein showed that the recombinant had not acquired any biological disadvantage due to modification of its genome structure by insertion of this gene.

## 4. Discussion

The classical ND vaccines have shown limited efficacy in controlling viral shedding and in overcoming high levels of MDA in chickens. To address these problems, several authors have proposed the use of HVT as a viral vector vaccine for the expression of the NDV F and HN proteins, due to its ability to persist in the presence of high MDA titers [10], to reduce viral shedding in vaccinated birds, and to induce long-lasting cellular and humoral immunity with an application of one single dose [10,12,15,16,17]. Moreover, the HVT genome can accommodate large fragments of foreign DNA in its genome without any interference to its replication [51]. In this study, we developed a recombinant HVT vector vaccine expressing the NDV F antigen (rHVT-F) from genotype XII, circulating in Peru, using the NHEJ–CRISPR/Cas9 and Cre-LoxP System. The rHVT-F vaccine administered in 1-day-old SPF chicks was able to induce high protection in the vaccinated chickens by the absence of any typical clinical signs of NDV disease, as evaluated at 14 dpc, generating complete protection against the challenge strain of NDV belonging to genotype XII at 49 dpv in all vaccinated chickens, compared to the non-vaccinated control group that presented 100% of mortality at 6 dpc by the route of the oculo-nasal challenge (a mimic field infection route). Nevertheless, in this study is truth that the challenge at 49 dpv does not reflect some situations on the field, where chickens would be exposed to NDV infections in earlier dpv. Therefore, would be necessary to evaluate the vaccine’s effectiveness with a challenge in earlier dpv in future studies.

Our results showed that the rHVT-F vaccine also stopped the shedding of the challenge virus from the upper respiratory tract at 5 dpc. Moreover, none of the vaccinated chickens shed the challenge virus in the cloacal swabs, indicating a complete suppression of the virus in the intestinal tract possibly due to the solid systemic and humoral immunity ensuring strong suppression of the challenge virus replication in the body of vaccinated chickens. These findings have been reported in other studies [10,12,15,17]. In this study, we achieved isolated the rHVT-F virus in CEF cells from PBLs samples at 28, 35, and 74 dpv, probably due to its replication in the lymphoid organs, which would cause a presumed systemic immunity. On the other hand, there is evidence that chickens vaccinated with Rispens, SB-1, and HVT have shown viral genome integration at the telomeres of chromosomes of the spleen due to harbors telomeric repeats (TMRs) in its viral genome [52], so this evidence could probably explain the presence of virus in PBLs in this study.

A recent study indicated that the viral shedding pattern is associated with the challenge virus inoculation route, wherein an inoculation by the intranasal route, the excretion of the challenge virus in the oro-nasal route is detectable between 100 and 90% of vaccinated chickens (20 and 28 days of age, respectively) at 4 dpc, showing a great susceptibility of the upper respiratory tract [17]. This can be explained by the rapid colonization of the virus, possibly due to an incomplete or weak local immune response of the mucosal surface of the respiratory tract in the first days of the disease onset [10]. However, as mentioned above, we did not detect the challenge virus shedding in 5 dpc in vaccinated chickens in the oral route.

On the other hand, is it possible that these results respond to the hypothesis about the greater efficacy of a homologous vaccine with a significant reduction in viral shedding? The truth is that, in this study, we hypothesized that this rHVT-F vaccine developed against an antigenically similar challenge strain would improve the efficacy of the vaccine with clinical protection and additionally contribute to a complete reduction in viral shedding which is shown in our results.

Some scientific studies have suggested that higher genetic distance between vaccine antigens and field challenge viral strains is one of the causes of the inefficient reduction in the challenge virus shedding in the respiratory tract of vaccinated birds [53]. On the other hand, others have reported that, considering that all NDV strains belong to one serotype Avian paramixovirus 1 (APMV-1), antigens from any NDV strain can be used as a vaccine for any genotype to provide efficient protection from morbidity and mortality against any virulent NDV challenge and that the efficacy of vaccines in controlling viral shedding could be mainly associated with the characteristic of the vaccines, route of application, inadequate and difficulties with vaccination, lack of maintaining the cold chain of vaccines, immune system, and different environmental conditions [53,54]. Nevertheless, due to increased viral diversity, all NDV strains are divided into two classes, Class I (single genotype) and class II (21 genotypes), based on a complete sequence analysis of the F gene [5], which potentially warrants the need to renew and develop antigenically matched vaccines that can give improved protection against velogenic NDV, decrease the shedding and transmission of the virus [55,56].

In chickens, the level of the systemic humoral response against NDV has been considered a good indicator of protection against the challenge. The IgM antibody is the predominant isotype produced after initial exposure to a novel antigen, but IgY antibody is the main isotype produced in the active secondary systemic response to infection following IgM. On the other hand, the main difference between chicken IgY and mammalian IgG is the longer H chain in the chicken molecule. Furthermore, IgY consists of five domains (V, C1–C4) different from IgG with four domains [57].

In this study, we monitored IgY antibody levels against NDV in sera by ELISA with titers that increased significantly at 34 (*p* = 0.0063) and 49 dpv (*p* = 0.0497). Although we did not monitor other Ig such as IgM or IgA, these antibodies could also play an essential role against the NDV challenge. Since one study has detected IgM, IgY, and IgA levels in serum samples after immunization with rHVT-F by in-house ELISAs, suggesting the induction of a complete humoral immune response [58].

On the other hand, we also obtained a positive antibody titer (4.3 ± 1.2 log2) by HI test at 49 dpv. Although this test is usually used to measure the concentration of anti-hemagglutinin-neuraminidase antibodies induced by conventional live vaccines, it is different for the vaccine in this study, where the HVT only expresses the F protein and not HN protein from NDV. Other authors have described that positive HI titers in vaccinated birds, with a vaccine vector that expresses the F protein, are associated with a steric hindrance effect in which many F antibodies are capable of inhibiting hemagglutination [59].

In this study, we also demonstrated that F gene insertion did not affect recombinant virus replication and morphology when compared to wtHVT. These results coincided with other work that used the NHEJ–CRISPR/Cas9 technique to generate HVT-VP2 [26,27]. On the other hand, the first-generation recombinant HVT viruses obtained using other systems tend to replicate more slowly when compared to the wtHVT, thus generating suboptimal levels of viral antigen expression, and this may contribute to a delay in the induction of an optimal level of protective antibodies. It could be due to multiple passages under antibiotic selection and several rounds of plaque purification that may alter the biological characteristics finally eliciting instability. These steps are eliminated or reduced with the CRISPR/Cas9 system due to its efficiency in integrating different foreign genes into the wtHVT genome. Various studies have shown that recombinant viruses obtained by the CRISPR/Cas9 system were stable in vitro [26,37,45,48]. In agreement with this report, we demonstrated that the inserted cassette remained stable even after 20 passages, as confirmed by PCR and IFA. We also demonstrated the genetic integrity of the F expression cassette by PCR.

Conventional recombination techniques have been used to develop the existing recombinant HVT–NDV vaccines. However, these techniques are time-consuming and laborious. Recently, several studies have reported the usefulness of CRISPR/Cas9 technology in studying specific genes and essential proteins of herpesviruses [44,46,47], thus establishing its use as an ideal tool for editing viral genomes [26,27].

Therefore, CRISPR/Cas9-based gene editing can be used as a fast, simple, and effective platform for the generation of new recombinant vaccines that express diverse antigens in the HVT genome [26,27,28,29,49]. This innovative gene-editing tool (CRISPR/Cas9) has rapidly established itself as a versatile and powerful tool that has enabled the insertion of foreign genes, such as red fluorescent protein (RFP) [26], GFP [44], infectious bursal disease virus (IBDV) VP2 antigen [26,27], infectious laryngotracheitis virus (ILTV) glycoprotein D-glycoprotein I (gDgI) [49], and avian influenza virus (AIV) HA antigen [28,29,49]. Based on the success of these studies, we used the CRISPR/Cas9 technique to generate a new HVT recombinant that expresses the NDV F protein from genotype XII, a specific genotype widely circulating in Peru. NDV has two immunogenic proteins: HN and F. We selected the F gene for the generation of the recombinant HVT, as other studies have demonstrated that it is sufficient to induce complete protection [9,10,12,17,60].

The molecular mechanisms to repair DSBs, created by Cas9, follow two pathways: NHEJ and HDR. We employed the NHEJ pathway to introduce the NDV F expression cassette into intergenic region UL45–UL46 because it occurs in actively cycling cells, and its efficiency is three to six-fold higher than that of HDR. The HDR pathway is considered less efficient since it is restricted to the S and G2 phases of the cell cycle. Another advantage of the NHEJ pathway is the capability to repair the DNA in 30 min, while HDR can require a delay of seven hours to complete the process [61].

Besides this, in the NHEJ pathway, we do not need to build homologous arms, making the cloning process more straightforward and faster. Corroborating our results, other colleagues determined that this is an efficient and fast pathway to generate knock-in of genes in the herpesvirus genome.

## 5. Conclusions

This is the first report on the development of a recombinant HVT vector vaccine candidate, expressing the NDV F antigen from the XII genotype circulating in Peru, using the NHEJ–CRISPR/Cas9 and Cre-LoxP systems capable of attributing complete protection in SPF chickens and reducing viral shedding against a genotype XII challenge. However, more studies are still needed, such as evaluating the cellular immune response and showing the protection in broiler chicken, but this is the first step to explore a new generation of vaccines, with antigens from circulating viruses, that could lead to better ND control in the future.

## Figures and Tables

**Table 1:** A list of the primer sequences used in this study.

Primers and sgRNAs	Sequences (5′→3′)
NDV-F(XII)-5F	TGGGAACAATACCCTCGATCA
HVT UL46-5R	GTTTCGGAATCTGGCAGGGT
HVT UL45F	TCGCAAACGCCAAAGTTCTG
HVT UL46R	CGAGCAATGACCCTCCAGTT
1F	CGTTGTAAAACGACGGCCAG
1R	TGGCTTGGGAACAATACCCT
2F	ATTGAGTCACCACCCCTATGC
2R	CCCAACTTCTCGGGGACTGT
sgRNA-UL45-46-F	CACCgTAGACATTATAAACATAATA
sgRNA-UL45-46-R	AAACTATTATGTTTATAATGTCTAc

**Table 2:** Frequency of isolation of challenge virus in vaccination groups.

Group	Number of Viral Shedding Chickens (Positive/Total)					
	3 dpc ^(a)^		5 dpc		8 dpc	
	Oral	Cloacal	Oral	Cloacal	Oral	Cloacal
Vaccinated	2/7	0/7	0/7	0/7	0/7	0/7
Control	7/7	5/7	6/6	6/6	NS ^(b)^	NS

^(a)^ dpc = days post-challenge. ^(b)^ NS = no survivors.

## Data Availability

Not applicable.

## Supplementary Materials

The following supporting information can be downloaded at: https://www.mdpi.com/article/10.3390/v14040793/s1, Figure S1: Plaque size comparison between wtHVT and rHVT-F virus.
