# Supplementary material for: A Recombinant Turkey Herpesvirus Expressing the F Protein of Newcastle Disease Virus Genotype XII Generated by NHEJ-CRISPR/Cas9 and Cre-LoxP Systems Confers Protection against Genotype XII Challenge in Chickens
Source: Viruses. 2022 Apr 11;14(4):793. doi: 10.3390/v14040793 (PMC9030537; doi:10.3390/v14040793)
Supplement: Supplementary file 1 [file viruses-14-00793-s001.zip › viruses-1620051-supplementary.pdf]

viruses

Supplementary Materials

A Recombinant Turkey Herpesvirus Expressing the F Protein of Newcastle Disease Virus Genotype XII Generated by NHEJ-CRISPR/Cas9 and Cre-LoxP Systems Confers Protection against Genotype XII Challenge in Chickens

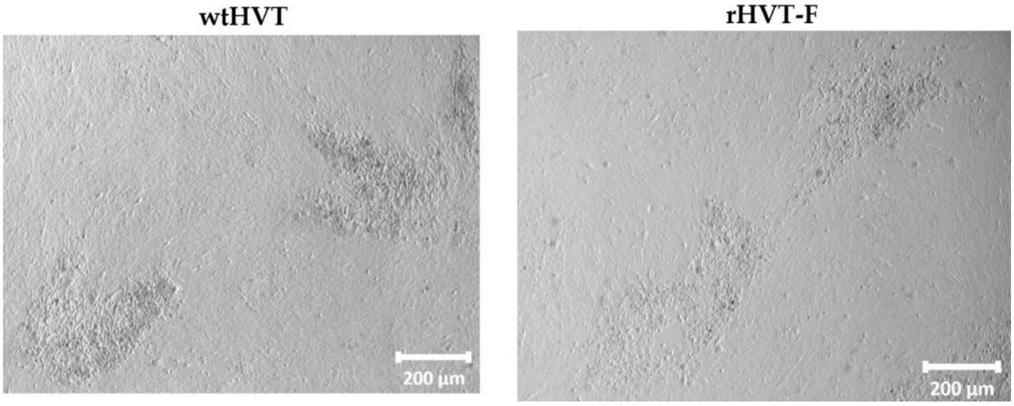

Figure S1. Plaque size comparison between wtHVT and rHVT-F virus. CEF cells were infected with the rHVT-F and wtHVT, then 5 days post-infection the plaques were observed by bright-field microscopy: 50× magnification, all bars represent 200 µm.
